# Exacerbated signs of an immunosuppressive AIDS-like disease in macaques infected with multiple retroviruses

**DOI:** 10.1186/1742-4690-8-S1-A207

**Published:** 2011-06-06

**Authors:** Jane L Mitchell, Simon Hood, Ghazi Auda, Neil M Almond, Nicola J Rose

**Affiliations:** 1 Division of Retrovirology, National Institute for Biological Standards and Control, South Mimms, Potters Bar, Hertford, EN6 3QG, UK; 2 Division of Virology, National Institute for Biological Standards and Control, South Mimms, Potters Bar, Hertford, EN6 3QG, UK

## 

In retroviral co-infection one or more of the viruses may display altered dynamics impacting on the pathology within the host. To characterise the nature of such changes we studied cynomolgus macaques naturally infected with three retroviruses. DNA was isolated from eleven tissues from macaques infected with SFV-1 (4), SRV-2/SFV-1 (4), STLV-1/SFV-1 (3), SRV-2/STLV-1/SFV-1 (6), and one uninfected macaque. Proviral load and distribution were evaluated by quantitative PCR. Immunopathology was assessed by H&E staining and immunohistochemistry, using antibodies against T-, B-lymphocytes and macrophages in the MLN and spleen.

Our data reveal that in co-infected macaques a significant increase in the SRV-2 proviral distribution and a trend towards an increase in the proviral load of SRV-2 but not STLV-I or SFV-1 occurs Pathological changes were more adverse in co-infected macaques, identified by the presence of nodular hyperplasia or, in extreme instances, follicular depletion. A greater number of B- and T-lymphocytes and macrophages were observed in co-infected macaques than in singly-infected macaques. The atypical distribution of the B- and T-cells is suggestive of altered immunopathology. Thus co-infection increases the distribution and proviral load of SRV-2 thereby creating an atypical immunopathology within the lymphoid organs which may enhance SRV-2 pathogenesis and hinder the host’s ability to control infection.

